# Expectant management of prolonged hemolysis following complete transcatheter coil closure of a patent ductus arteriosus after previous pulmonary artery banding: a case report

**DOI:** 10.1186/s12872-021-02365-z

**Published:** 2021-11-17

**Authors:** Mao-Sheng Hwang, Ching-Chia Kuo, Hung-Tao Chung, Hsin-Mao Hsu, Jaw-Ji Chu, Chao-Jan Wang

**Affiliations:** 1 Department of Pediatrics, Chang Gung Memorial Hospital, Linkou Medical Center, Chang Gung University College of Medicine, 5-7, Fu-Shin Street, Kweishan, Taoyüan, 333 Taiwan; 2 Department of Surgery, Chang Gung Memorial Hospital, Linkou Medical Center, Chang Gung University College of Medicine, Taoyüan, Taiwan; 3 Department of Radiology, Chang Gung Memorial Hospital, Linkou Medical Center, Chang Gung University College of Medicine, Taoyüan, Taiwan

**Keywords:** Case report, Coil, Hemolysis, Patent ductus arteriosus, Pulmonary artery banding, Therapeutic embolization, Transcatheter

## Abstract

**Background:**

Transcatheter coil occlusion has been the treatment of choice for closure of small patent ductus arteriosus (PDA). In spite of its safety, complications such as hemolysis still occasionally occur. And the hemolysis almost always occurs following partial transcatheter closure of PDA; hence, it occurs extremely rarely following complete transcatheter closure of PDA without residual ductal flow.

**Case presentation:**

Here, we describe a male newborn who developed prolonged hemolysis following complete transcatheter coil closure of his PDA after previous palliative pulmonary artery banding. Over the following days, we corrected his refractory anemia by repeated blood transfusion with packed red blood cells and frequently monitored his hemoglobin, serum total bilirubin, and serum lactate dehydrogenase. We speculated that the high-velocity pulmonary blood flow jet coming into contact with the extruded part of the coil led to red blood cell mechanical injury, thereby resulting in the hemolysis. We adopted expectant management in expectation of the endothelialization of the coil with a resultant reduction in the hemolysis. The hemolysis, as expected, was reduced gradually until it spontaneously resolved 81 days after coil implantation.

**Conclusions:**

This case reminds us that hemolysis can still potentially occur following complete transcatheter coil closure of PDA. It also highlights the importance of preventing coils from extruding into the pulmonary artery in patients after previous pulmonary artery banding.

**Supplementary Information:**

The online version contains supplementary material available at 10.1186/s12872-021-02365-z.

## Background

Transcatheter coil occlusion has been the treatment of choice for closure of small patent ductus arteriosus (PDA). In spite of its safety, complications such as hemolysis still occasionally occur [1]. And the hemolysis almost always occurs following partial transcatheter closure of PDA [1]; hence, it exceedingly rarely occurs following complete transcatheter closure of PDA without residual ductal flow [2]. In one study, the authors presumed that aortic blood contact with the extruded part of the coil (Flipper detachable coil, Bloomington, Indiana, USA) led to red blood cell (RBC) mechanical injury, thereby resulting in hemolysis [2]. We recently treated a unique male newborn who, unexpectedly, developed prolonged hemolysis following complete transcatheter coil closure of his PDA after previous palliative pulmonary artery banding. To our knowledge, this is probably the second case of hemolysis occurring following complete transcatheter closure of PDA, with likely sharing a similar mechanism with the first patient but occurring in a different scenario.

## Case presentation

**Fig. 1:**
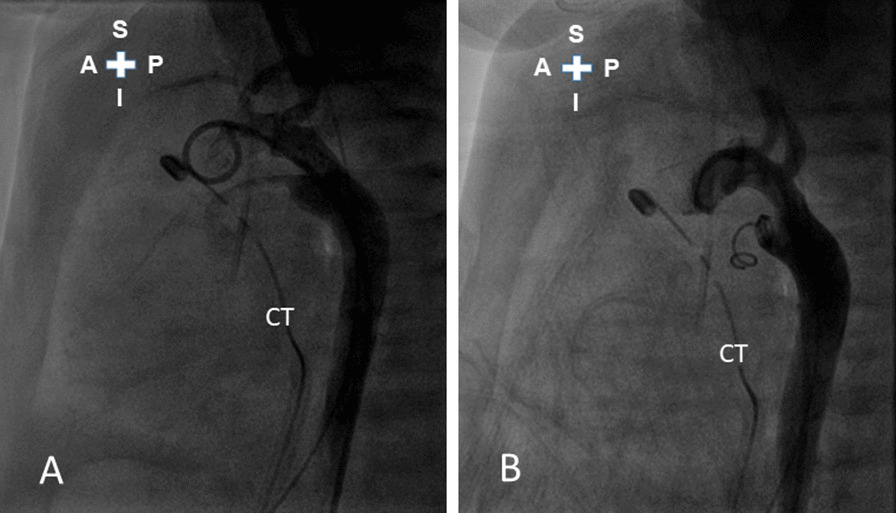
**a** Lateral-view aortogram showed a Krichenko Type A1 patent ductus arteriosus with the narrowest portion 1.7 mm in diameter; **b** Lateral-view aortogram after the implantation of a Gianturco coil (MWCE-38-6-4) showed complete closure of the patent ductus arteriosus without residual shunting, but approximately half of the coil was extruded into the pulmonary artery with a resultant redundant and bulky appearance. *A* anterior; *CT* chest tube; *I* inferior; *P* posterior; *S* superior

A 1-day-old full-term male newborn with birth body weight 2930 gm was diagnosed with a double-outlet right ventricle {S, D, D} with a subpulmonary ventricular septal defect (Taussig-Bing anomaly) and a right aortic arch associated with a right-sided PDA. He underwent palliative pulmonary artery banding through left minithoracotomy when he was 20 days old, but the PDA was not ligated at that time due to its right-sided position. The postoperative Doppler echocardiography showed peak velocity of the systolic jet across the main pulmonary artery band was 4.0 m/s, which indicated the estimated peak pressure gradient across the band was 64 mmHg. However, the heart failure in the newborn was still uncontrollable despite adequately performed and hemodynamically effective pulmonary artery banding. Thus, transcatheter occlusion of the PDA (Krichenko Type A1, 1.7 mm at its narrowest potion) (Fig. 1a) was performed via a retrograde route using a Gianturco coil (MWCE-38-6-4, Cook, Inc., Bloomington, IN) on the 25th day of life. Postimplantation aortography showed no residual shunting, but approximately half of the coil was extruded into the pulmonary artery with a resultant redundant and bulky appearance (Fig. 1b).

**Fig. 2:**
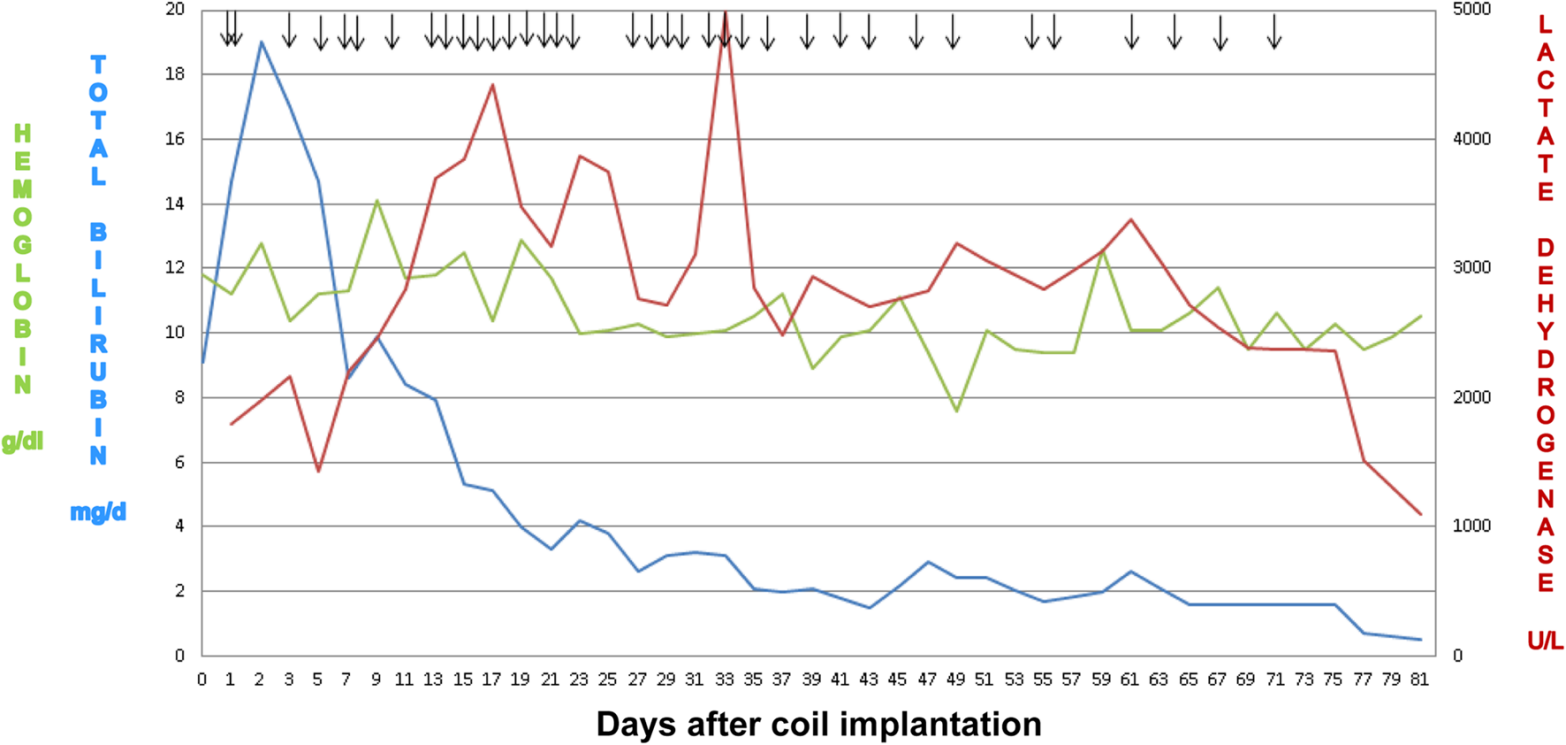
Temporal changes in hematological and biochemical parameters noted after coil closure of patent ductus arteriosus.↓, denoting blood transfusion

**Fig. 3:**
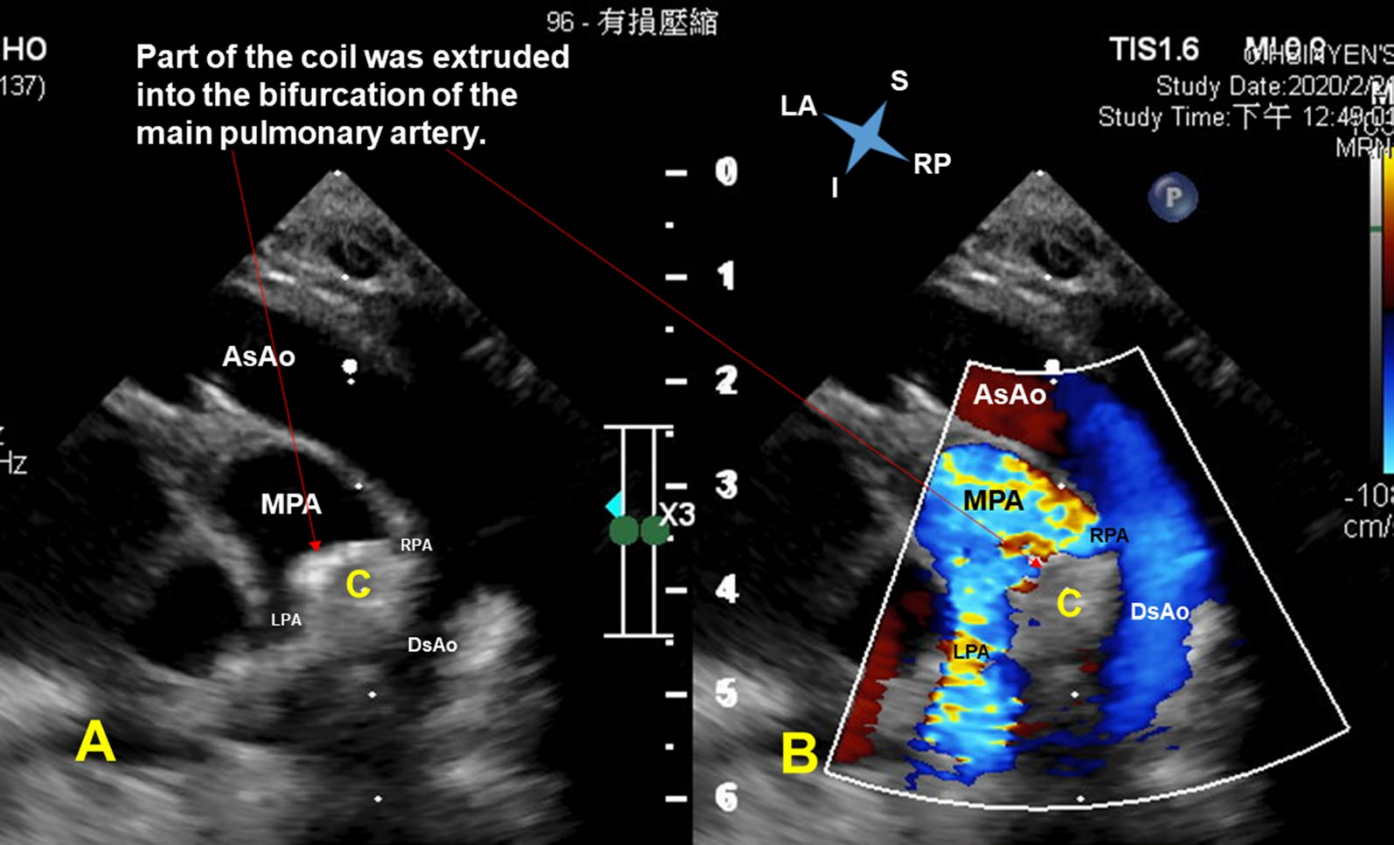
Suprasternal long-axis view with the transducer rotated and oriented to the right scapular tip (simultaneous 2-dimensional [**a**] and color flow Doppler [**b**] recordings) revealed right aortic arch without residual ductal shunting, but part of the coil was extruded into the bifurcation of the main pulmonary artery, in addition to the high-velocity mosaic pulmonary flow jet resulting from the previously performed pulmonary artery banding. It also showed the high-velocity pulmonary flow jet hit the extruded coil. *AsAo* ascending aorta; *C* coil; *DsAo* descending aorta; *I* inferior; *LA* left-anterior; *LPA* left pulmonary artery; *MPA* main pulmonary artery; *RP* right-posterior; *RPA* right pulmonary artery; *S* superior

**Fig. 4:**
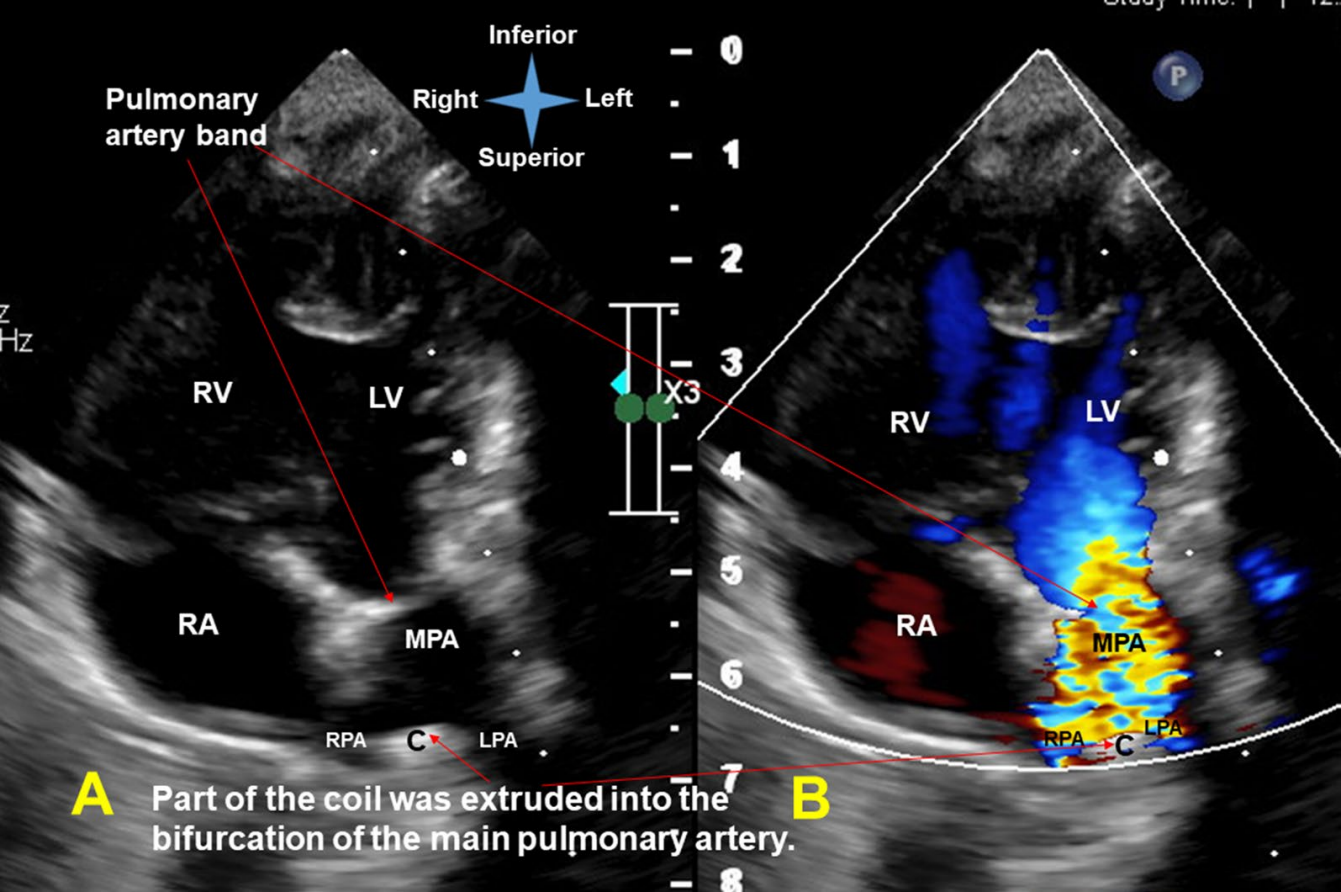
Modified apical five-chamber view (simultaneous 2-dimensional [**a**] and color flow Doppler [**b**] recordings) showed no residual ductal shunting, but part of the coil was extruded into the bifurcation of the main pulmonary artery, in addition to the high-velocity mosaic pulmonary flow jet resulting from the previously performed pulmonary artery banding. It also showed the direct collision of the high-velocity pulmonary flow jet with the extruded coil. *C* coil; *LPA* left pulmonary artery; *LV* left ventricle; *MPA* main pulmonary artery; *RA* right atrium; *RPA* right pulmonary artery; *RV* right ventricle

**Fig. 5:**
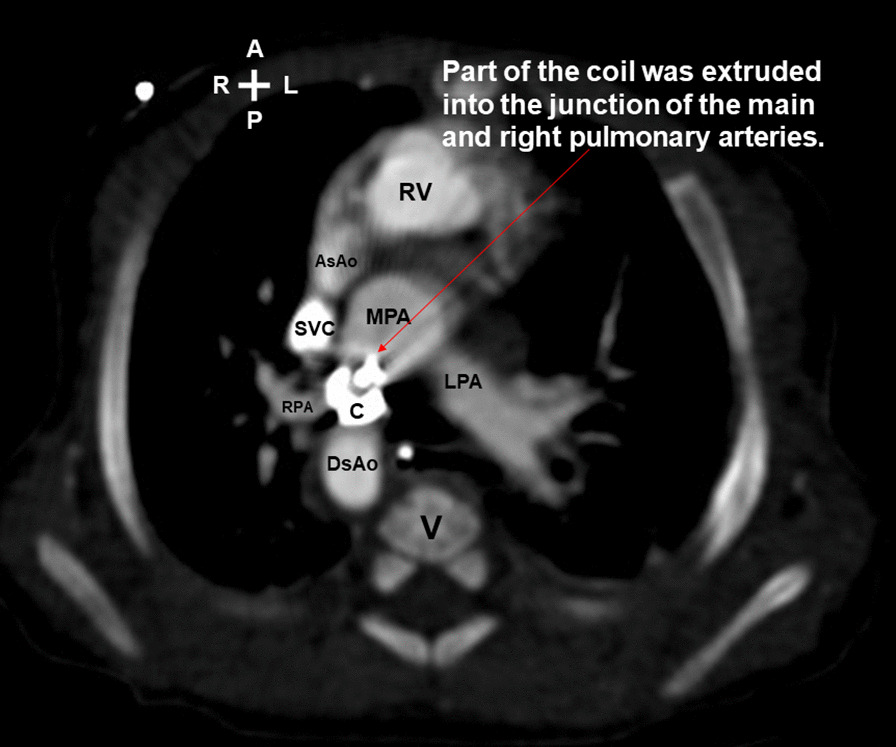
Computed tomography angiography (axial view) more clearly demonstrated that part of the coil was extruded into the junction of the main and right pulmonary arteries. *A* anterior; *AsAo* ascending aorta; *C* coil; *DsAo* descending aorta; *L* left; *LPA* left pulmonary artery; *MPA* main pulmonary artery; *P* posterior; *R* right; *RPA* right pulmonary artery; *RV* right ventricle; *SVC* superior vena cava; *V* vertebra

**Fig. 6:**
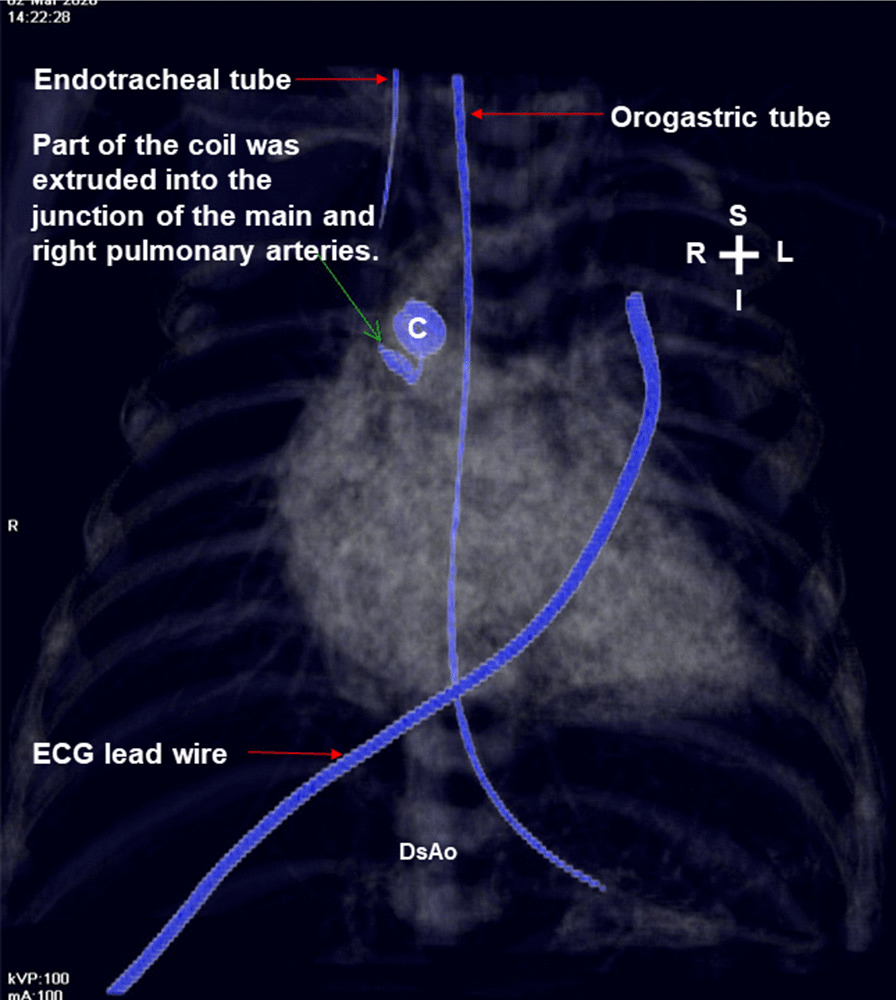
Frontal image of merged 3-dimentional chest computed tomography with transparency and 3-dimentional volume rendering of metallic coil more clearly revealed that part of the coil was extruded into the junction of the main and right pulmonary arteries. *C* coil; *DsAo* descending aorta; *ECG* electrocardiography; *I* inferior; *L* left; *R* right; *S* superior

**Fig. 7:**
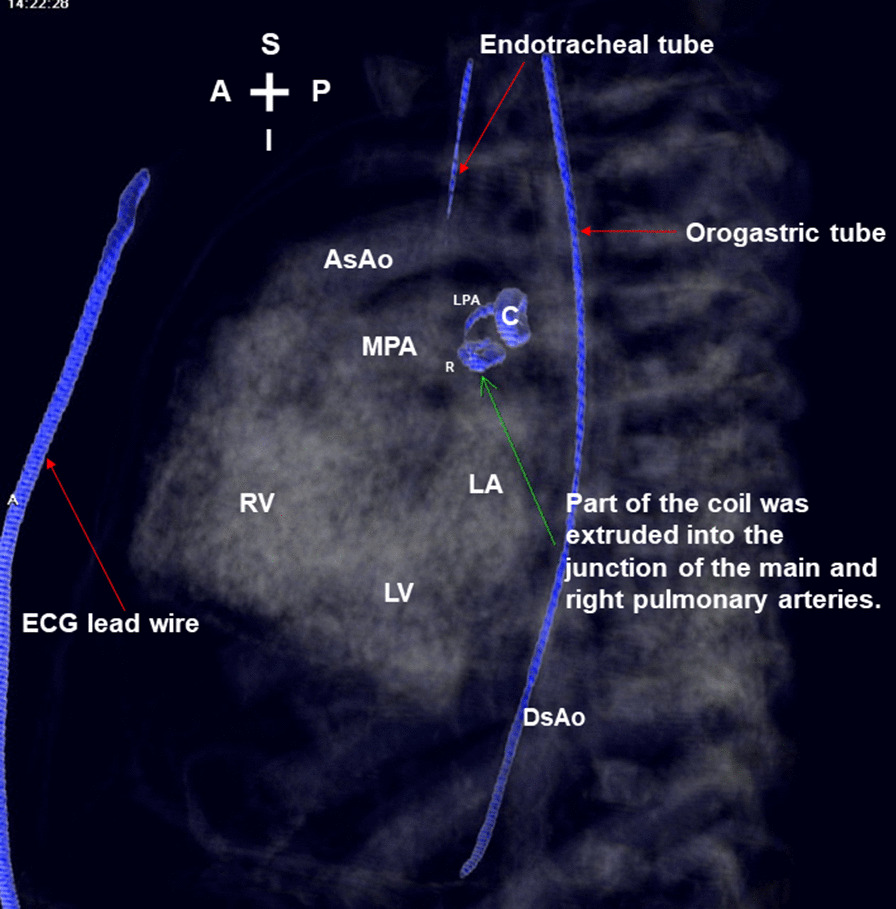
Sagittal image of merged 3-dimentional chest computed tomography with transparency and 3-dimentional volume rendering of metallic coil also more clearly showed that part of the coil was extruded into the junction of the main and right pulmonary arteries. *A* anterior; *AsAo* ascending aorta; *C* coil; *DsAo* descending aorta; *ECG* electrocardiography; *I* inferior; *LA* left atrium; *LPA* left pulmonary artery; *LV* left ventricle; *MPA* main pulmonary artery; *P* posterior; *R* right pulmonary artery; *RV* right ventricle; *S* superior

Unexpectedly, he developed gross hematuria 3 h later. The laboratory data 1 day after coil implantation revealed elevated serum total bilirubin (from 9.1 to 14.7 mg/dl; normal range 0.1–1.2 mg/dl), increased serum lactate dehydrogenase (1796 U/L; normal range 135–260 U/L), decreased hemoglobin (from 11.8 to 11.2 g/dl; normal range 9.6–12.8 g/dl) (Fig. 2) and reduced serum haptoglobin (< 7.69 mg/dl; normal range 30–200 mg/dl). The postimplantation echocardiography showed no residual ductal shunting, but part of the coil was extruded into the bifurcation of the main pulmonary artery, in addition to the high-velocity mosaic pulmonary flow jet resulting from the previously performed pulmonary artery banding. It also showed the high-velocity pulmonary flow jet hit the extruded coil. (Fig. 3 and Additional file 1: Video 1; Fig. 4 and Additional file 2: Video 2). The postimplantation computed tomography angiography more clearly demonstrated that part of the coil was extruded into the junction of the main and right pulmonary arteries (Figs. 5, 6, 7). We did not know the exact cause of the suspected hemolysis at this time. Over the following days, we were only able to correct his refractory anemia by repeated blood transfusion with packed RBCs and frequently monitor his hemoglobin, serum total bilirubin, and serum lactate dehydrogenase (Fig. 2).

At day 24 postimplantation, reticulocytosis (reticulocyte count 5.4%; normal range 0.4–2.8%) was noted. A peripheral blood smear showed schistocytes and fragmented RBCs. Both direct and indirect Coombs tests were negative. Thus, intravascular nonimmune hemolysis was more definitively diagnosed. After review of a previous similar case report [2] and the postimplantation images (Figs. 3, 4, 5, 6, 7 and Additional file 1: Video 1; Additional file 2: Video 2), we speculated that a high-velocity pulmonary blood flow jet coming into contact with the extruded part of the coil led to RBC mechanical injury, thereby resulting in the hemolysis [2, 3]. The high-velocity pulmonary blood flow jet resulted from the previously performed pulmonary artery banding.

At that time, we decided to adopt expectant management in expectation of the endothelialization of the coil with a resultant reduction in the hemolysis [4]. Fortunately, the degree of hemolysis began decreasing gradually until spontaneous resolution 81 days postimplantation, as reflected in the gradual reduction in serum total bilirubin and lactate dehydrogenase and a reduced need for blood transfusion (total amount of transfused packed RBCs: 1127 ml/36 transfusions) (Fig. 2). The patient then underwent Jatene procedure at 111 days of age (day 86 postimplantation). Unfortunately, he expired 9 h postoperatively due to low cardiac output syndrome after the extracorporeal membrane oxygenation support was removed.

Timeline of the relevant events is summarized in Table 1.

**Table 1:** Timeline of relevant events

Age	Relevant events
1 D/O	Diagnosed with a double-outlet right ventricle {S, D, D} with a subpulmonary ventricular septal defect (Taussig-Bing anomaly) and a right aortic arch associated with a patent ductus arteriosus
20 D/O	Pulmonary artery banding performed
25 D/O	Transcatheter coil occlusion of the patent ductus arteriosus; hematuria developing 3 h later
26 D/O	Hemolysis suspected and start of repeated blood transfusion to correct its refractory anemia
49 D/O	Intravascular nonimmune hemolysis more definitively diagnosed
96 D/O	Stop of blood transfusion
106 D/O	Spontaneous resolution of hemolysis presumed
111 D/O	Jatene procedure performed
112 D/O	Expired after the extracorporeal membrane oxygenation support removed

*D/O* days old

## Discussion and conclusions

Herein, we describe a case of prolonged hemolysis following complete transcatheter coil closure of PDA after previous palliative pulmonary artery banding. We speculate that a high-velocity pulmonary blood flow jet coming into contact with the extruded part of the coil led to RBC mechanical injury. Thus, the underlying mechanism of hemolysis in this patient was probably similar to that in the aforementioned case, although the authors of that study did not further elaborate on the possible underlying mechanisms [2].

The possible underlying mechanisms of hemolysis in our patient might be extrapolated from those that occur after mitral valve repair [5]. The following two mechanisms are probably involved: direct collision of the high-velocity pulmonary blood flow jet with the coil and/or fragmentation of the high-velocity pulmonary blood flow jet by the coil [5]. These two mechanisms are associated with high shear stress, and the high-velocity pulmonary blood flow jet hitting the nonendothelialized coil surface might generate such stress, leading to traumatic disruption of the RBCs [5].

The possible clinical course of this rarely occurring hemolysis has not been well established, although the hemolysis in the previous similar case still persisted 47 days postimplantation before rescue surgery [2]. Prosthetic materials usually become rapidly endothelialized within several weeks [4, 5], and the degree of hemolysis presumably will be gradually reduced as the coil gradually becomes endothelialized [4]. However, the high-velocity pulmonary blood flow jet might denude the endothelium [5], thereby delaying the endothelialization process and hence prolonging the hemolytic course to the 81st day postimplantation.

With regard to potentially better management strategies of our patient, we retrospectively consider that the earlier percutaneous pulling of a large part of the coil grasped with a snare from the aortic end of the ductus away from the pulmonary artery to the aortic end of the ductus, leaving approximately 1 loop of the coil in the pulmonary end of the ductus, might have shortened the duration of hemolysis. The potential benefit of this attempt is that it might prevent the high-velocity pulmonary blood flow jet from coming into contact with the coil, thereby avoiding the direct collision of the high-velocity pulmonary blood flow jet with the coil and/or fragmentation of the high-velocity pulmonary blood flow jet by the coil [5]. If this attempt succeeds, the hemolysis probably could resolve more quickly and, in addition, it could avoid the much invasive rescue surgery as described in the first case [2]. The potential risk of this attempt is that pulling of too large part of the coil away from the pulmonary artery to the aortic end of the ductus might result in embolization of the coil, aortic coarctation, or hemolysis as described in the first case [2]. But strict adherence to the method of leaving approximately 1 loop of the coil in the pulmonary end of the ductus and avoiding pulling through the ductus might prevent its embolization [6]. Moreover, the space of the aortic ampulla of the ductus of this particular patient is large enough to accommodate the large part of the coil without causing aortic coarctation or hemolysis (Fig. 1a, b).

But prevention is better than cure. Then, what could be done to prevent this hemolytic complication? We think that strict adherence to the method of allowing 1 loop of the coil to form in the pulmonary artery during the first step of deploying the coil is of paramount importance [6]. With this 1 loop formed in the pulmonary artery, the catheter with pusher wire and the partially extruded coil all together are withdrawn until the exposed loop of the coil touches the pulmonary end of the ductus [6]. During the following maneuver of deploying the remainder of the coil into the aortic end of the ductus, the pulmonary loop of coil is observed closely to be sure that the single loop of coil in the pulmonary end of the ductus remains in the proper location and is not pulled through the ductus or pushed back into the pulmonary artery [6]. This method should prevent the coil from extruding into the pulmonary artery, thereby avoiding the hemolytic complication.

In conclusion, this case reminds us that hemolysis can still potentially occur following complete transcatheter coil closure of PDA, and we should prevent the coil from extruding into the pulmonary artery in patients who had previously undergone pulmonary artery banding or other similar scenarios that resulted in the high-velocity pulmonary blood flow jet. It also demonstrates the protracted course of hemolysis with the potential for spontaneous resolution; earlier rescue strategies could probably markedly shorten the duration of hemolysis.

## Supplementary Information

**Additional file 1: Video 1.** Suprasternal long-axis view with the transducer rotated and oriented to the right scapular tip (simultaneous 2-dimensional [**right**] and color flow Doppler [**left**] recordings) revealed right aortic arch without residual ductal shunting, but part of the coil was extruded into the bifurcation of the main pulmonary artery, in addition to the high-velocity mosaic pulmonary flow jet resulting from the previously performed pulmonary artery banding. It also showed the high-velocity pulmonary flow jet hit the extruded coil.

**Additional file 2: Video 2.** Modified apical five-chamber view (simultaneous 2-dimensional [**right**] and color flow Doppler [**left**] recordings) showed no residual ductal shunting, but part of the coil was extruded into the bifurcation of the main pulmonary artery, in addition to the high-velocity mosaic pulmonary flow jet resulting from the previously performed pulmonary artery banding. It also showed the direct collision of the high-velocity pulmonary flow jet with the extruded coil.

## Data Availability

All data generated or analyzed during this study are included in this published article.

## Abbreviations

PDA: Patent ductus arteriosus
RBC: Red blood cell
